# Supplementary material for: Chemical modulation of transcriptionally enriched signaling pathways to optimize the conversion of fibroblasts into neurons
Source: eLife. 2019 May 17;8:e41356. doi: 10.7554/eLife.41356 (PMC6524968; doi:10.7554/eLife.41356)
Supplement: Figure 1—source data 1. [file elife-41356-fig1-data1.pdf]

Age (yr)	ID	Full Name	Sex	Fibroblast Source	Fibroblast RNAseq/ MethylCHIP Passage	Used to Generate Figures
65	L1	14096	M	ADRC	NA	Fig2D,E | Fig 4C,F,I | Fig 3 - Fig Sup 4 | Fig 5 - Fig Sup 1 | Fig 3 - Fig Sup 2
0	L2	BJ CRL-2522	M	Coriell	p13	Fig 1E,G,H | Fig 3 | Fig 4K | Fig 4 - Fig Sup 1 | Fig 3 - Fig Sup 6 | Fig 3 - Fig Sup 2| Fig 5 - Fig Sup 1
1	L3	AG08498	M	Coriell	p16	Fig 5
65	L4	8150	M	ADRC	NA	Fig 4L | Fig 4 - Fig Sup 1 | Fig 3 - Fig Sup 6 | Fig 3 - Fig Sup 2 | Fig 3 - Fig Sup 4 | Fig 2 - Fig Sup 4
29	L5	ERF1	F	Erlangen	p14	Fig 1E,G,H | Fig 2 D,E | Fig 3 | Fig 4C | Fig 5 | Fig 3 - Fig Sup 5
29	L6	AG04054	M	Coriell	p12	Fig 4A, Fig 4E | Fig 5
71	L7	UKERfO3H-X-	M	Erlangen	p11	Fig 1E,G,H | Fig 3 | Fig 5 | Fig 3 - Fig Sup 5
88	L8	3158LG	M	ADRC	NA	Fig 4C | Fig 3 - Fig Sup 6 | Fig 3 - Fig Sup 2 | Fig 3 - Fig Sup 4 | Fig 2 - Fig Sup 4
84	L9	3383	M	ADRC	p17	Fig 2D,E | Fig 5 | Fig 3 - Fig Sup 6
1	L10	AG08498	M	Coriell	p9	Fig 2A,B | Fig 4L | Fig 4 - Fig Sup 1 | Fig 5 - Fig Sup 1
