# Supplementary material for: Chemical modulation of transcriptionally enriched signaling pathways to optimize the conversion of fibroblasts into neurons
Source: eLife. 2019 May 17;8:e41356. doi: 10.7554/eLife.41356 (PMC6524968; doi:10.7554/eLife.41356)
Supplement: Figure 2—source data 2. [file elife-41356-fig2-data2.pdf]

Chemical (Abbreviation)	Pathway	Inhibitor/Activator	Effect on iN	IC50 (uM)	Supplier	Reference
Pyrintegrin (PY)	Integrin Signaling	Activator	+	2	Tocris	Xu, Yue et al. (2010)
CK666 (CK)	Integrin Signaling	Inhibitor	-	4	Sigma	Burke, Thomas A., et al. (2014)
PamCSK4 (PM)	JAK1, JAK2 and TYK2 Interferon Sig	Activator	-	12	Tocris	Manukyan, Maria, et al. (2005)
Pf3758309 (PF)	JAK1, JAK2 and TYK2 Interferon Sig	Inhibitor	+	0.0187	Cayman	Murray, Brion W., et al. (2010)
SU6656 (SU)	Epithelial Adherens Junction	Inhibitor	-	0.28	Cayman	Woodcock, Simon A., (2009)
Angiotensin II (AN)	Epithelial Adherens Junction	Activator	=	0.0015	Tocris	Suzuki, Yusuke, et al. (2003)
KC7F2 (KC)	HIF1α Signaling	Inhibitor	+	15	Tocris	Narita, Takuhito, et al. (2009)
ML228 (ML)	HIF1α Signaling	Activator	-	1.5	Tocris	Theriault, Jimmy R., (2012)
Demethylasterriquinone B1 (DM)	IGF-1 Signaling	Activator	-	100	Sigma	Webster, Nicholas JG, Kaapjoo Park, and Michael C. Pirrung. (2003)
AZ960 (AZ)	IGF-1 Signaling	Inhibitor	+	0.002	Cayman	Gozgit, Joseph M., (2008)
Ehop-016 (EH)	Actin Nucleation by ARP-WASP	Inhibitor	-	1.1	Selleckchem	Montalvo-Ortiz, Brenda L., et al. (2012)
PI3 Kinase act (PI)	Actin Nucleation by ARP-WASP	Activator	+	0.65	SC Biotech	Noh, Min Young, et al. (2013)
IPA3 (IP)	Rho-Family GTPase	Inhibitor	=	2.5	Tocris	Murakoshi, Hideji, Hong Wang, and Ryohei Yasuda. (2011)
ZM336372 (ZM)	Rho-Family GTPase	Activator	+	0.07	Tocris	Van Gompel, Jamie J., et al. (2005)
pkg inhibitor (PK)	Sertoli Junction Signaling	Activator	-	86	Cayman	Wexler, Eric M., Patric K. Stanton, and Scott Nawy. (1998)
U46619 (U4)	Sertoli Junction Signaling	Inhibitor	-	0.032	Cayman	Reese, Jeff, et al. wall. (2009)
syk inhbitor (SY)	Remodeling of Epithelial Adherens	Inhibitor	=	0.005	Cayman	Lai, Justine YQ, et al. (2003)
NSC87877 (NS)	Remodeling of Epithelial Adherens	Activator	-	0.355	Cayman	Song, Mina, et al. (2009)
dabigatram (DB)	Intrinsic Prothrombin Activation	Inhibitor	=	0.0045	Cayman	Ebner, Thomas, Klaus Wagner, and Wolfgang Wienen. (2010)
Thrombin TRAP6 (TR)	Intrinsic Prothrombin Activation	Activator	-	0.8	Tocris	Rudroff, Claudia, et al. (1998)
